# Functional analysis of Pro-inflammatory properties within the cerebrospinal fluid after subarachnoid hemorrhage in vivo and in vitro

**DOI:** 10.1186/1742-2094-9-28

**Published:** 2012-02-08

**Authors:** Ulf C Schneider, Jennifer Schiffler, Nahid Hakiy, Peter Horn, Peter Vajkoczy

**Affiliations:** 1 Department of Neurosurgery, Charité Universitaetsmedizin, Augustenburger Platz 1, 13353 Berlin, Germany; 2 Center for Stroke Research Berlin, Charité Universitaetsmedizin, Berlin, Germany

**Keywords:** Subarachnoid hemorrhage, Neuro-inflammation, Cerebral vasospasm, Delayed brain injury, Secondary brain injury

## Abstract

**Background:**

To functionally characterize pro-inflammatory and vasoconstrictive properties of cerebrospinal fluid after aneurysmal subarachnoid hemorrhage (SAH) in vivo and in vitro.

**Methods:**

The cerebrospinal fluid (CSF) of 10 patients suffering from SAH was applied to the transparent skinfold chamber model in male NMRI mice which allows for in vivo analysis of the microcirculatory response to a superfusat. Microvascular diameter changes were quantified and the numbers of rolling and sticking leukocytes were documented using intravital multifluorescence imaging techniques. Furthermore, the pro-inflammatory properties of CSF were assessed in vitro using a monocyte transendothelial migration assay.

**Results:**

CSF superfusion started to induce significant vasoconstriction on days 4 and 6 after SAH. In parallel, CSF superfusion induced a microvascular leukocyte recruitment, with a significant number of leukocytes rolling (day 6) and sticking (days 2-4) to the endothelium. CSF of patients presenting with cerebral edema induced breakdown of blood vessel integrity in our assay as evidenced by fluorescent marker extravasation. In accordance with leukocyte activation in vivo, significantly higher in vitro monocyte migration rates were found after SAH.

**Conclusion:**

We functionally characterized inflammatory and vasoactive properties of patients' CSF after SAH in vivo and in vitro. This pro-inflammatory milieu in the subarachnoid space might play a pivotal role in the pathophysiology of early and delayed brain injury as well as vasospasm development following SAH.

## Background

Cerebral vasospasm following acute aneurysmal subarachnoid hemorrhage (SAH) is still one of the most feared complications of this disastrous disease. Although recent advances in vasospasm treatment have been reported [1,2], the pathophysiological mechanisms of this entity are still under investigation. Within the past few years, a shift of paradigm with respect to the impact of cerebral vasospasm has been initiated. The theory that cerebral vasospasm is the only cause of delayed brain injury in patients after SAH is being increasingly questioned and hypotheses of other mechanisms contributing to early or delayed brain damage are being discussed [3].

Recently, an activation of inflammatory pathways has been suggested to be involved in the pathogenesis of secondary brain injury after SAH [3]. For example, perivascular immune complex accumulation could be shown around the large conductance vessels as signs for inflammatory processes [4,5]. The theory of inflammation to play a role in the pathophysiology of SAH had been addressed before, suggesting e.g. the arachidonic acid metabolism with its successive metabolites prostaglandins, prostacyclins, thromboxans and leukotrienes to be potential targets [6-14]. Those changes could be observed within the first few days after SAH, days before clinical or diagnostic evidence for vasospasm existed. Based on this, a relationship between inflammation, and cerebral vasospasm as well as delayed brain injury after SAH has benn discussed [15]. Yet, a functional analysis of post-SAH CSF might give further insight in the underlying mechanisms, as well as the time course of inflammation and vasospasm development, and provide new therapeutic targets or diagnostic tools.

Therefore, this study was conducted to evaluate potential vasoconstrictive as well as pro-inflammatory properties of cerebrospinal fluid (CSF) after SAH. For this purpose we established an in vivo model in which vascular reactivity as well as leukocyte endothelial interaction could be observed after exposition to post-SAH CSF. We furthermore applied a cellular in vitro assay to confirm inflammatory cell attraction by post-SAH CSF.

## Methods

Animal experiments were approved by the responsible ethics committee and conducted according to the regulatory guidelines (Regierungspräsidium Karlsruhe). The withdrawal of post-SAH CSF from the critically ill patients was approved by the local ethics committee and conducted throughout the routine CSF sampling (Landesamt für Gesundheit und Soziales Berlin). All diagnostic assessments were done on the basis of our institutional guidelines.

### Patient management and assessment of cerebral vasospasm

For the present study, 10 consecutive patients with severe SAH (WFNS Grade III or worse, Fisher Grade 3) were included. The screening was done at the initial presentation in our institution. The patient characteristics are displayed in Table 1. All patients received an external ventricular drainage (EVD) for measurement and therapy of elevated intracranial pressure (ICP) according to the clinical guidelines.

**Table 1:** Patient characteristics.

				Vasospasm		
**Age**	**WFNS**	**Ay location**	**TCD**	**DSA**	**Xe-CT**	**Timepoint**

52	4	aCom	no	yes	n.d.	3

74	4	n.a.	no	no	n.d.	n.a.

64	4	aCom	yes	yes	no	7

56	4	aCom	yes	yes	yes	7

61	4	aCom	yes	n.d.	yes	9

60	4	aCom	yes	yes	yes	7

50	5	ICA	yes	n.d.	n.d.	n.a.

62	4	aCom	yes	yes	yes	9

44	3	pCom	yes	yes	yes	7

42	5	ICA	yes	yes	yes	4

Vasospasm diagnostics were done according to a multimodal concept. The screening methods were judged positive for: TCD > 200 cm/s; DSA > 50% narrowing of at least one conductance artery; Xenon CT < 31 ml/100 g/min. The timepoint of diagnosis establishment is indicated in days after SAH

Abbreviations: *aCom *anterior communicating artery; *pCom *posterior communicating artery; *ICA *internal carotid artery; *TCD *transcranial doppler; *DSA *digital subtraction angiography; *Xe-CT *Xenon CT; *n.a*. not applicable; *n.d*. not done; *WFNS *World Federation of Neurological Surgeons Grading

**Figure 1:**
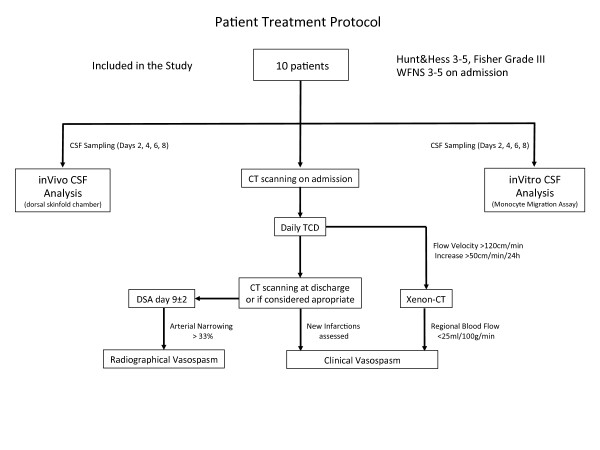
**Study protocol showing standard patient management, timepoints and decision making of imaging as well as the further processing of post-SAH CSF**.

Daily bilateral transcranial Doppler ultrasound was performed for vasospasm screening (blood flow velocity in the middle cerebral artery (MCA)). When blood flow velocity increased over 120 cm/min or by more than 50 cm/min within 24 h, Xenon-cCT was scheduled to evaluate cerebral blood flow with good spatial resolution and to assess the hemodynamic relevance of the suspected vasopasm. Furthermore, digital subtraction angiography (DSA) was performed according to our standard protocol on days 7-9 after SAH (or earlier in case of suspected vasospasm). The diagnosis of radiographical vasospasm was made on the basis of DSA, documenting angiographic arterial narrowing. The diagnosis of clinical vasospasm was defined by clinical signs of ischemia and/or vasospasm-associated infarctions in the CT, as well as a relevant territorial hypoperfusion in Xe-CT. TCD served as screening method only. In repeated native CT-scans cerebral infarctions and edema development were documented (Figure 1).

As a control group six consecutive patients were assessed, who were referred to our department for myelography diagnostics of lumbar spine degeneration. These were considered healthy individuals in terms of CSF alterations (no high grade spinal canal stenosis). Although almost all of these patients had medical side conditions due to their age (arterial hypertension, hyperlipidemia, cardiac disease etc.), inflammatory issues were ruled out by standard laboratory blood sampling.

### Transparant chamber preparation

The transparent dorsal skinfold chamber in the mouse is a model for studying microvascular function under physiological and pathophysiological conditions [16-18]. Recently, this in vivo assay has been used in order to study pro-inflammatory and vasoactive properties of different molecules which were directly applied to the preparation. Following superfusion/incubation the response of the microvascular bed to the superfusat was anaylzed by intravital multiflourescence videomicrcoscopy [19-21].

Briefly, mature immune competent NMRI mice were anaesthetized using an intraperitoneally applied mixture of ketamine and xylazine (7.5 mg ketamine and 2.5 mg xylazine/100 mg body weight). The hair on the back of the mice was carefully shaved and chemically epilated. The dorsal skin was elevated and implanted into two symmetrically applied titanium frames. In this way the extended double layer of skin was trapped. The titanium frames spared an area of 15 mm in diameter in which the upper skin layer was completely removed and the remaining layer (muscle, subcutaneous tissue and epidermis) was covered with a glass slide.

In this way direct, non-invasive access to the vasculature was gained for the superfusion experiments and repeated multifluorescence videomicroscopy. For the experimental protocol only chambers without signs for bleeding or inflammatory processes (edema, vasodilation or stasis) were included. In parallel, a catheter was placed into the jugular vein and was subcutaneously guided to the dorsal end of the skinfold chamber. This catheter was used for application of the fluorescent dyes.

### Intravital multifluorescence videomicroscopy

For multifluorescence intravital videomicroscopy, the animals were immobilized in a special mouse restrainer and fixed upon a table with the titanium frame of the skinfold chamber. The microscopic examination was conducted after intravenous application of 1 ml of 5% Fluorescein Isothiocyanate (FITC)-Dextrane (Mr 150.000; Sigma Chemical Co.; St.Louis, MO). The leucocytes were labelled by 0.1 ml of 0.2% rhodamine 6 G (Mr = 489; Serva Feinbiochemica GmbH & Co.; Heidelberg, Germany). The chamber was observed using a Zeiss fluorescence stereo-microscope equipped with a high pressure mercury lamp for epi-illumination (I 2/3 bluefilter 450-490 nm; N2 greenfilter 530-560 nm). By using two dyes with different emission spectrum the process of multi-step leukocyte recuitment, i.e. rolling, sticking, and migration along the endothelium (Rhodamine 6 G) as well as microhaemodynamics (FITC-Dextrane) could be evaluated sequentially in the according observation fields by switching between the filters. A CCD videocamera mounted on the microscope allowed for recording of the data for later off-line analysis.

The experimental observation began with a mapping of the microvascular bed of the preparation for later re-assessment of the regions of interest. Five arterioles and five venules (diameter 20-60 μm) were randomly selected for the documentation. The quantitative analyses of the microcirculation were performed using a 10× long distance-objective as well as a 20× immersion-objective with magnifications of 200× and 400× respectively.

### Experimental protocol of in vivo experiments

At the time of initial patient recruitment, the skinfold chamber was prepared. For each patient and for each respective time point one mouse was prepared (total number of animals used for our 16 patients: 64). To exclude influence of the anaesthesia or the operation trauma the skinfold chamber experiments did not start before day 2 after preparation. On days 2, 4, 6 and 8 after occurrence of the SAH, 3-5 ml CSF were harvested from the EVD of each respective patient. The sample was centrifuged at 3000/min for 10 min and the supernatant was withdrawn. All samples were used freshly for the superfusion experiments, without storage or freezing. One patient who showed signs of bacterial meningitis was excluded from the protocol after diagnosis on days 4.

First, FITC-Dextran and rhodamine6G were injected i.v. and a baseline analysis of the microvasculature was performed. 5 distinct pre-capillary arterioles and post-capillary venules (20-60 μm) were identified for further analysis of microhemodynamics and leukocyte/endothelial-interaction. Approximately 30 min later, after removal of the cover glass, the skinfold chamber was superfused with 0.5-1 ml of post-SAH- or control CSF for 10 min. Following superfusion, the chamber was closed again with a fresh glass cover and the microvasculature was visualized using the multifluorescence videomicroscopic setup. The following microhemodynamic parameters were documented quantitatively in the same microvascular segments before and 10, 30, 60, 120, 180 and 240 min after superfusion with CSF: microvascular diameter, erythrocyte velocity and microvascular permeability, the later of which was defined via the extent of extravasation of the low-molecular weight fluorescent marker Rhodamine 6 G. For the evaluation of the leukocyte flow properties, the leukocyte/endothelial interaction was divided into temporarily ('rolling') and permanently adhesive ('sticking') leucocytes. Rolling was defined as a passage time of more than 20 s within the vascular segment (normalized for the diameter). Permanent adhesion was documented for sticking leukocytes that remained in one place for longer than 20 s. The latter were counted as cells per square micrometer. The area was calculated from the diameter and the length of the vessel segment (200 μm).

### In vitro monocyte migration assay

The membrane surfaces of trans-well chambers (pore size 5 μm) were coated with 50 μl 0.1% gelatine, were covered with a layer of human umbilical cord endothelial cells (HUVEC, 80.000/200 μl) and incubated for two days at 37°C. Staining with crystal violet (1%) permitted observation of the integrity and the confluence of the cell layer twice during the incubation time course. In a second step, 24 h before starting the assay, the human acute monocytic leukemia cell line THP-1 was incubated with TGF beta-1 (stock solution 5 μg/ml, working solution 10 ng/ml) and vitamin D (stock solution 50 μmol, working solution 50 nmol) for 24 h. After stimulation these cells yielded a strong expression of CD14+ on their surface, which was verified by FACS analysis, supposing that they had differentiated into mature monocytes.

After washing of the HUVEC cell layer, a total number of 400000/200 μl BSA (0.5%) was applied to each trans-well chamber and 600 μl of post SAH CSF was applied to the respective wells. Again, the CSF samples of patients undergoing myelography for spinal diagnostics served as controls. After incubation at 37°C for 90 min the assay was stopped and the total number of CD14+ cells was counted that had transmigrated through the HUVEC cell layer.

### Statistical evaluation

The statistical analysis of arteriolar diameter, rolling and sticking leukocytes and transmigrated monocytes was done using univariate ANOVA with posthoc Bonferoni correction. Edema/Rhodamine Correlation was evaluated using Wilcoxon ChiSquare test. *P*-values smaller than 0.05 were considered statistically significant.

## Results

Eight patients out of 10 developed hemodynamically, angiographically or clinically accessible cerebral vasospasm. The diagnosis of cerebral vasospasm was established 6.6 ± 2.1 days after the onset of the bleeding.

### Post-SAH CSF induces vasoconstriction earlier than the clinical manifestation of vasospasm

**Figure 2:**
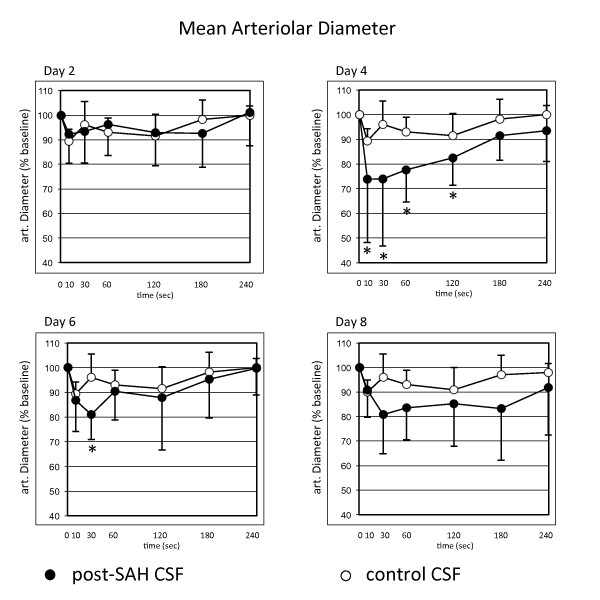
**Quantitative analysis of the arteriolar diameter after superfusion of the dorsal skinfold chamber with cerebrospinal fluid (CSF) of patients after severe subarachnoid haemorrhage**. Lower diameters are documented after superfusion with post-SAH CSF. Significantly lower diameters are documented on days 4 and 6 after SAH. Values are given as means ± standard deviation. * = *p *< 0.05 vs. baseline values. N = 10 for post-SAH group (days 2 and 4) and 9 (days 6 and 8), respectively, due to exclusion of one patient's CSF after diagnosis of bacterial meningitis. N = 6 for control group.

The quantitative analysis of the CSF samples revealed a significant vasoconstrictive potential of the post-SAH CSF when compared to control-CSF on days 4 and 6 (Figure 2). The maximum vasoconstriction occurred on day 4, even before clinical or radiographical diagnosis of vasospasm. Remarkably, vasoconstrictions of up to 100% could be repeatedly shown in different arteriolar segments of preparations superfused with CSF from patients with vasospasm.

### Post-SAH CSF becomes highly pro-inflammatory within 2 days after SAH

**Figure 3:**
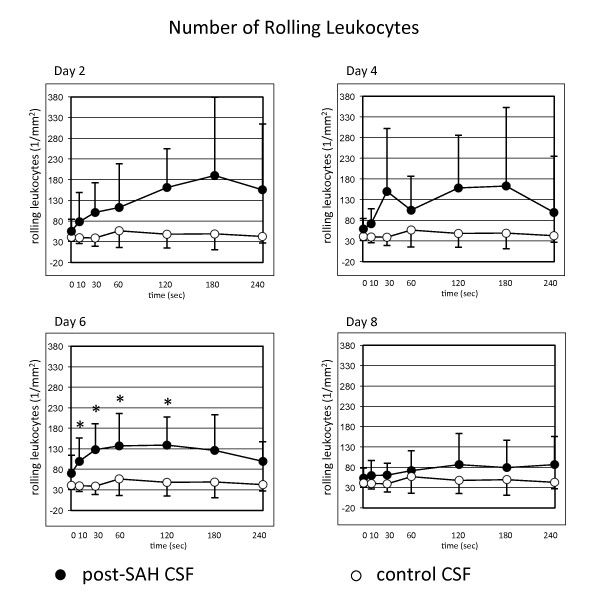
**Quantitative analysis of rolling leukocytes after superfusion of the dorsal skinfold chamber with post-SAH CSF showing significantly higher numbers of rolling leukocytes on day 6**. Values are given as means ± standard deviation. * = *p *< 0.05 vs. baseline values. N = 10 for post-SAH group (days 2 and 4) and 9 (days 6 and 8), respectively, due to exclusion of one patient's CSF after diagnosis of bacterial meningitis. N = 6 for control group.

**Figure 4:**
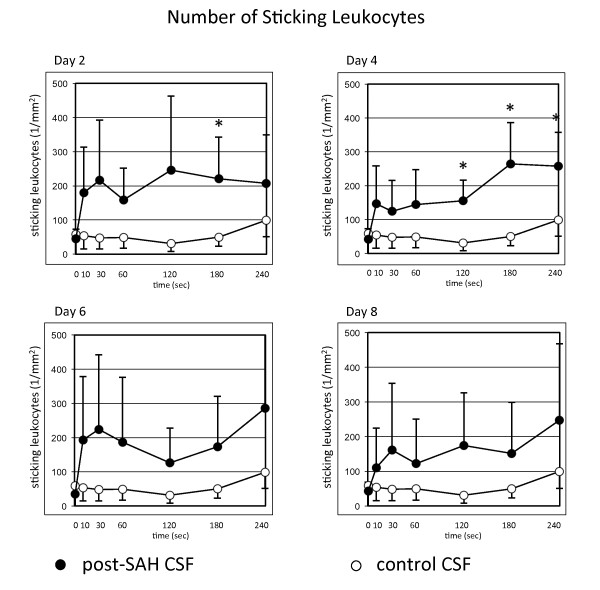
**Quantitative analysis of sticking leukocytes after superfusion of the dorsal skinfold chamber with post-SAH CSF showing significantly higher numbers of sticking leukocytes on days 2 and 4**. Values are given as means ± standard deviation. * = *p *< 0.05 vs. baseline values. N = 10 for post-SAH group (days 2 and 4) and 9 (days 6 and 8), respectively, due to exclusion of one patient's CSF after diagnosis of bacterial meningitis. N = 6 for control group.

Long before clinical manifestation of cerebral vasospasm, analysis of the CSF-samples revealed significant pro-inflammatory properties of post-SAH CSF. Starting on day 2 after SAH, an activation of the leukocyte/endothelial interaction with an increased number of recruited leukocytes, both rolling and sticking fractions, could be shown. A significantly increased number of rolling leukocytes could be shown on day 6, the number of sticking leukocytes was significantly elevated from day 2 through day 4 (Figures 3 and 4).

**Figure 5:**
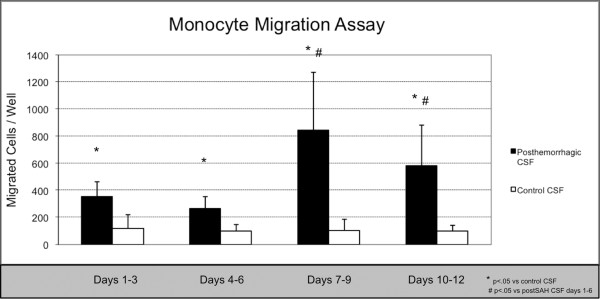
**The monocyte transendothelial migration assay showed significantly higher numbers of CD14+ monocyte cells that had migrated through the HUVEC cell layer after stimulation with post-SAH CSF when compared to stimulation with control CSF**. * = *p *< 0.05 vs control CSF # = *p *< 0.05 vs days 1-3 and days 4-6. N = 10 for post-SAH group (days 1-3) and 9 (days 4-12), respectively, due to exclusion of one patient's CSF after diagnosis of bacterial meningitis. N = 6 for control group.

To further assess the pro-inflammatory properties of post-SAH CSF in vitro we established a THP-1 monocyte transendothelial migration assay. The total number of CD14+ cells that migrated through the HUVEC cell layer was significantly elevated after stimulation with post-SAH CSF during the entire 12 days observation period after SAH (Figure 5). Again, a significant difference could be observed much earlier than the clinical manifestation of cerebral vasospasm, confirming the results obtained for our in vivo analysis.

### Post-SAH CSF of patients with cerebral edema breaks down microvascular integrity

On days 6 and day 8 after SAH an increase of the microvascular permeability was documented in terms of extravasation of the low-molecular fluorescent marker Rhodamine G6 from the microvasculature. This extravasation could be predominantly observed in the postcapillary, venular segments of the microcirculation. A correlation with the clinical course of the individual patients showed that CSF-induced leakiness of blood vessels was associated with the development of brain edema and cerebral infarctions (Table 2).

**Table 2:** An increased extravasation of Rhodamine 6 G was documented in the preparations' microvasculature after superfusion with CSF of patients in whom edema or stroke were present at the according time points

		Extravasation of Rhodamine 6G		
**Edema/Stroke**		**positive**	**negative**	
	
	present	5	2	
	
	absent	0	3	*p *< 0.05

## Discussion

In this study we functionally characterized the pro-inflammatory and vasoactive milieu in post-SAH CSF in vivo and in vitro. We described two assays in which the vasoconstrictive and inflammatory properties of human CSF could be evaluated in i) a well-established in vivo animal model for microcirculatory studies and ii) an in vitro monocyte transendothelial migration assay. This experimental setup provided evidence for significant early vasoconstrictive and inflammatory characteristics of human post-SAH CSF. This was displayed by a significant arteriolar vasoconstriction and intravascular leukocyte recruitment in the in vivo assay, that preceded the diagnosis of cerebral vasospasm in our patients. The increased chemotaxis of THP-1 monocytes in the in vitro assay documented upregulated trans-endothelial migration properties of inflammatory cells upon stimulation with post-SAH CSF.

The involvement of inflammatory reactions in early brain injury, development of cerebral vasospasm and delayed brain injury after SAH has been more and more discussed within the past years [3,6-15,22]. Hypotheses exist that inflammatory processes also occur in the walls of ruptured aneurysms and might therefore facilitate or lead to aneurysmal bleeding [23-25]. However, the mediation of these inflammatory processes still remains unclear. Clinically relevant therapeutic targets have not yet been established.

Supposing that post-SAH CSF has the capability to attract a significant number of inflammatory cells from the circulation and pool them in the subarachnoid space, an inflammatory milieu might be elicited along the blood vessels and in the basal cisterns. This inflammatory environment might initiate or contribute to cerebral vasospasm. As a possible mediator for vasoconstrictive and inflammatory activity interleukin-6 has already been discussed. An upregulation of this inflammatory cytokine has been observed extraparenchymally in the CSF, but also in the brain parenchyma itself (microdialysis) in patients after SAH [26]. In addition, our group has demonstrated that monocytes within the subarachnoid space may be the source of vascoactive/vasoconstrictive mediators such as endothelin-1 [27]. To further enlight the underlying mechanisms, the two described assays add favourably to the existing literature, which mostly focused on histological, molecular or ex vivo analysis of inflammatory changes following SAH [6-14].

Our study has several limitations. Due to the small number of patients, a correlation analysis of the detected CSF changes with the development of cerebral vasospasm or secondary brain injury could not be performed. Nevertheless, our data were suggestive for a time relationship between the occurrence of vasoconstriction and leukocyte activation in the assays and the clinical onset of vasospasm. Cerebral vasospasm was diagnosed in our patients between days 3 and 9 by TCD, Xe-CT and/or DSA (mean 6.6 ± 2.1 days), which is well in accordance with the known time course of vasospasm in man. Yet, despite the tight diagnostic regime, we cannot exclude, that we missed an earlier development of cerebral vasospasm in our patients and the observed CSF alterations were an epiphenomenon. Furthermore, the evaluation of the dorsal skinfold chamber elicited high standard deviations, due to a high interindividual variability, especially in the leukocyte/endothelial interaction. We therefore faced the following statistical problems. 1. Irregularities in the data curves gave an impression of an unsteady response to the superfusion. 2. Alpha as well as beta errors might be underestimated.

Of 10 patients eight developed cerebral vasospasm, which seems much. Yet, all of the patients, included in our study suffered from severe SAH (WFNS III or worse, Fisher Grade 3). Thus, the observed high incidence of morphological vasospasm is within the reported range. Furthermore, we used a tight regimen of multiple testing methods, not to miss out on the occurrence of cerebral vasospasm.

## Conclusion

The present study supports the existing theory that inflammatory mechanisms can contribute to secondary brain injury after SAH. We could functionally demonstrate an inflammatory millieu in post-SAH CSF in vivo and in vitro. The detected inflammatory changes were accompanied by microvascular diameter changes, and preceded the clinical diagnosis of cerebral vasospasm in our patient population.

## Competing interests

Source of Funding of the project: Centre for Stroke Research, Berlin, Germany. Otherwise, none of the authors has any financial or non-financial competing interests concerning the current study.

## Authors' contributions

UCS: conduction of the experiments, analysis and interpretation of the data, composition of the manuscript (full responsibility for the content). JS: conduction of experiments, interpretation of the data, involved in composition of the manuscript (partial responsibility for the content). NH: establishment of the technical procedures, conduction of experiments, review of the manuscript (partial responsibility for the content). PH: substantial contributions to concept and design, conduction of experiments, interpretation of data (partial responsibility for the content). PV: conception and design, interpretation of the data, revision and approval of the manuscript (full responsibility for the content). All authors read and approved the final manuscript.
